# Advanced efficient energy management strategy based on state machine control for multi-sources PV-PEMFC-batteries system

**DOI:** 10.1038/s41598-024-58785-2

**Published:** 2024-04-05

**Authors:** Badreddine Kanouni, Abd Essalam Badoud, Saad Mekhilef, Mohit Bajaj, Ievgen Zaitsev

**Affiliations:** 1 Automatic Laboratory of Setif, Electrical Engineering Department, University of Setif 1, Setif, Algeria; 2 School of Software and Electrical Engineering, Swinburne University of Technology, Melbourne, Australia; 3 Department of Electrical Engineering, Graphic Era (Deemed to be University), Dehradun, 248002 India; 4 Hourani Center for Applied Scientific Research, Al-Ahliyya Amman University, Amman, Jordan; 5 Graphic Era Hill University, Dehradun, 248002 India; 6 Applied Science Research Center, Applied Science Private University, Amman, 11937 Jordan; 7 Department of Theoretical Electrical Engineering and Diagnostics of Electrical Equipment, Institute of Electrodynamics, National Academy of Sciences of Ukraine, Peremogy, 56, Kyiv-57, 03680 Ukraine

**Keywords:** Renewable energy management, State machine control (SMC), Multi-source renewable system (MSRS), Photovoltaic cells (PV), Proton exchange membrane fuel cell (PEMFC), Energy efficiency, Load optimization, Energy science and technology, Engineering, Mathematics and computing

## Abstract

This article offers a PV-PEMFC-batteries energy management strategy (EMS) that aims to meet the following goals: keep the DC link steady at the standard value, increase battery lifespan, and meet power demand. The suggested multi-source renewable system (MSRS) is made to meet load demand while using extra power to fill batteries. The major energy source for the MSRS is photovoltaic, and fuzzy logic MPPT is used to guarantee that the PV operates at optimal efficiency under a variety of irradiation conditions. The suggested state machine control consists of 15 steps. It prioritizes the proton exchange membrane fuel cell (PEMFC) as a secondary source for charging the battery when power is abundant and the state of charge (SOC) is low. The MSRS is made feasible by meticulously coordinating control and power management. The MSRS is made achievable by carefully orchestrated control and electricity management. The efficacy of the proposed system was evaluated under different solar irradiance and load conditions. The study demonstrates that implementing the SMC led to an average improvement of 2.3% in the overall efficiency of the system when compared to conventional control techniques. The maximum efficiency was observed when the system was operating under high load conditions, specifically when the state of charge (SOC) was greater than the maximum state of charge (SOCmax). The average efficiency achieved under these conditions was 97.2%. In addition, the MSRS successfully maintained power supply to the load for long durations, achieving an average sustained power of 96.5% over a period of 7.5 s. The validity of the modeling and management techniques mentioned in this study are confirmed by simulation results utilizing the MATLAB/Simulink (version: 2016, link: https://in.mathworks.com/products/simulink.html) software tools. These findings show that the proposed SMC is effective at managing energy resources in MSRS, resulting in improved system efficiency and reliability.

## Introduction

The global consumption of electrical energy has experienced substantial growth in recent times^1,2^. In current scientific studies, the initial and predominant source of electricity generation has historically been fossil fuels and their byproducts, accounting for approximately 78–80% of the overall energy output^3,4^. Nevertheless, it is essential to acknowledge that the availability of these sources is limited, and the primary sources at hand will eventually be depleted, rendering them inadequate for our long-term requirements^5^. Furthermore, the necessity of electricity in the transportation industry has never been more obvious or pressing^6,7^. According to the International Energy Agency (IEA), transportation consumes 72.1% of all petroleum products (IEA), and when burnt, it usually causes anthropogenic climate change^8^. Also, in light of the substantial surge in the global population and escalating energy demands, there is a prevailing shift towards the adoption of novel and environmentally sustainable sources, including solar, wind, and hydrogen^9,10^. These provide several benefits to both humans and the environment when compared to conventional energy sources like heavy oil, coal, and natural gas; these alternatives are more sustainable and emit less CO_2_^11,12^. Renewable energy resources (RES) will take on a greater role in energy production. Of all renewable energy resources, solar energy is widely regarded as the foremost form of renewable energy source (RES) technology currently available due to its wide distribution, freedom from cost, and ecological friendliness^13,14^. Photovoltaic (PV) systems are experiencing a surge in popularity in both developing and developed nations^15,16^. While these technologies are improving in many ways, the negative aspects associated with them, such as their high investment cost, continue to be significant barriers to their widespread adoption^17,18^. However, hydrogen presents itself as a viable and sustainable energy carrier, with the potential to utilize solar electric energy generated by photovoltaic (PV) panels for water electrolysis^19,20^. This method of hydrogen production exhibits the advantage of emitting no carbon dioxide (CO_2_) during the process^21–23^.

Currently, researchers are contemplating the implementation of clean hybrid renewable energy systems (CHRES), which are anticipated to gain significant attention in the near future^24,25^. These systems aim to generate 100% renewable energy by utilizing technologies such as the hybrid photovoltaic fuel cell-storage system and solar-powered electricity generation^26,27^. In addition, the design of the hybrid system must consist of two renewable sources or more, and the efficiency of power generation systems is widely recognized, and RES is widely recognized as being among the most effective choices available^28,29^. Nevertheless, the reliance of renewable energy on weather patterns and its intermittent nature hinders its ability to provide a consistent supply of electricity^30,31^. Furthermore, the incorporation of various forms of renewable energy, among them wind and solar systems, results in significant production of electrical energy^32,33^. After a substantial duration of development, a substantial corpus of scholarly research and illustrative schematics concerning photovoltaic and battery DC microgrids and photovoltaic, wind, and battery DC microgrids have been generated^34,35^. However, it is important to note that the power output of PV and wind generators exhibit significant fluctuations in response to prevailing climatic conditions^36,37^. Due to battery performance and size limitations, this type of DC microgrid is incapable of meeting long-term energy needs^38,39^. To address the difficulty, the fuel cell is gaining popularity owing to its capability for long-term generating systems. Simultaneously, the photovoltaic panels function as a primary power source, while the batteries serve as a system for storing power for a relatively brief duration. The hybrid direct current (DC) microgrid, which includes PV generation, fuel cell systems, a battery system, and domestic loads, has garnered increasing interest among researchers^40^. The primary advantage of an HES system is its ability to be self-sufficient during varying climatic circumstances because it is not dependent on a single source. HES can sometimes be linked to the power grid or operated as a stand-alone microgrid^41,42^. Furthermore, the advantage of the combination of more than one renewable source than a conventional hybrid system (CHS) like a diesel generator with batteries is largely to save fossil fuels while also reducing their influence on the environment. However, the CHS are costly to run and maintain, particularly in rural places. Furthermore, they have a negative influence on the planet. In contrast, HES systems, which are more ecologically benign and sustainable, are the reason why hybrid energy systems based on renewable sources have garnered a significant amount of attention in the last few years^43,44^.

With the increasing use of DC components, many academics have turned their attention to the development of energy management techniques^45^. Several scholars have proposed such strategies, which can broadly be classified into two types: rule-based management strategies and optimization-based management strategies^46,47^. Rule-based management techniques can further be categorized into two types: deterministic rule-based EMS, exemplified by filters control (FBC) and wavelet transform, and state machine control (SMC)^48–50^. The other type of EMS is fuzzy rule-based, which includes standard fuzzy, adapting fuzzy, and adaptive neuro-fuzzy inference^51–53^. Research into fuel cells and hybrid power systems, such as the photovoltaic-fuel cell production system with batteries, has also been investigated, as discussed in reference^54,55^. In reference^56^, the author proposes an experimental validation of a combination of two renewable systems (photovoltaic/PEMFC/electrolyzer/batteries). In reference^57^, a novel energy management strategy for a microgrid consisting of photovoltaic (PV), wind, and battery systems was introduced. This strategy incorporates an intelligent prediction algorithm to optimize the utilization of these renewable energy sources. A hybrid energy management method is being used to improve the functioning of PV/FC/supercapacitor-battery renewable systems, which was discussed in Ref.^58^. The suggested single DC/DC converter operates in one way for the three sources of power (PV and FC) but in two ways for the battery bank. Despite the findings validating the suggested converter’s strong performance at the transitory and stable state operation functioning of the loads and PV irradiation step change, Among the drawbacks of the approach is that if there is an issue with the converter, the system will fail and the same design has been discussed in the Ref.^59,60^. In Ref.^61^, they proposed an energy management system consisting of PV/fuel cell/battery validated with Arduino. The authors in this Ref ^62^ proposed novel energy management in hybrid electric vehicle applications for a mixed system.

In Ref.^63^, the researcher provides the design of a battery charging circuit through intelligent MPPT using an SPV system. The aim of this study is to operate the designed solar photovoltaic (SPV) system at the maximum power point (MPP) under different environmental conditions. This improves efficiency, reduces overall system cost, and achieves the appropriate voltage and current for effective battery charging. Doing so aims to minimize battery losses and enhance the system’s life cycle. The authors in^64^ proposed the design of a robust multi-rating battery charger for the charging stations of electric vehicles via solar PV systems. The design of a robust multi-rating battery charger for charging electric vehicle stations via solar PV systems has been proposed. The goals of this study are to optimize the use of the proposed PV array using the I-P&O MPPT scheme in order to improve the efficiency of the system, lower the cost of the system, and minimize its complexity. In order to minimize battery losses and improve the lifespan of hybrid electric vehicles (HEVs)^65,66^, a comprehensive review of fuel cell-based topologies and multi-input DC–DC power converters for hybrid electric vehicles this study offers a comprehensive understanding of the subject matter for researchers and engineers engaged in this discipline.

The literature review focuses on the current obstacles associated with the integration of renewable energy, specifically in relation to photovoltaic (PV) systems and hydrogen fuel cells. Despite some progress, significant challenges related to system efficiency, energy storage, and grid integration continue to impede the widespread adoption of renewable energy sources. Photovoltaic systems (PV), due to their reliance on sunlight, encounter challenges related to variability and intermittency, which can negatively impact stability and reliability, particularly during periods of high demand or unfavorable weather conditions. Energy storage technologies, such as batteries, have limitations in effectively managing this variability due to constraints in energy density, efficiency, and lifespan. Grid operators face the challenge of managing intermittent renewable generation and ensuring grid stability, which requires the use of advanced forecasting and control techniques. The significant upfront capital requirements and limited governmental backing further hinder the widespread acceptance of renewable energy sources. To tackle these challenges, it is necessary to employ inventive energy management strategies and make progress in storage technologies, grid management, and policy frameworks. This will guarantee smooth integration into the global energy mix.

In this study, we proposed an energy management system for MSRS depending on the objective of the stabilization of the DC bus’s stability and the need for the load demand. The primary source of energy for the system is the photovoltaic array. The backup power source is comprised of a secondary battery and a fuel cell. The amount of excess PV electricity that is stored in batteries depends on the status of the power demand. Also, the battery is promoted to deliver power, and the fuel cell has two objectives: the first one works as a backup system if the PV system and the battery do not generate enough power for the load, and the second one charges the battery when the SOC is low. The strategy effectively integrates these two technologies to tackle the challenges of energy variability, grid stability, and system efficiency, offering a novel energy management approach. In this study, we have introduced an SMC approach that relies on 15 states. Our enhanced method incorporates more probabilities than previous research publications in the field, which have only nine cases^58^. The aim is to ensure robustness, simplicity in design, and ease of implementation. Furthermore, this technology may extensively advance hybrid power systems like microgrids.

This research examines the detrimental impact of imbalanced and uniform irradiance alteration, as well as the rapid change in load demand, on the output choices of SMC energy management. Next, we assess the precision of transitioning between different energy management modes in response to varying levels of sunlight and changing power requirements for photovoltaic, proton exchange membrane fuel cell (PV PEMFC) systems and battery outputs. In the end, the contribution to this paper is:

1. Regulation: it is necessary to maintain the voltage level to guarantee the system’s stability.
2. Load following refers to the need to meet load demand, particularly the crucial load, to ensure satisfaction.

This study offers practical insights into the implementation and real-world performance of the proposed energy management strategy through detailed simulations and performance evaluations. Finally, this research emphasizes the significance of regulatory assistance and policy modifications in facilitating the extensive implementation of renewable energy technologies. This study seeks to provide information to policymakers, industry stakeholders, and researchers and contribute to the ongoing discussion on sustainable energy solutions by highlighting these crucial aspects.

The following section of this article will focus on the proposed Multi-source renewable system in “Configuration of the proposed hybrid system”. In “Suggested state machine energy management strategy”, the state machine control for the MSRS is presented; “Results and discussions” presents the findings and debate of the proposed energy management under irradiation changes and various values of State of charge; and “Conclusion and future research directions” concludes with a conclusion and perspective.

## Configuration of the proposed hybrid system

**Figure 1:**
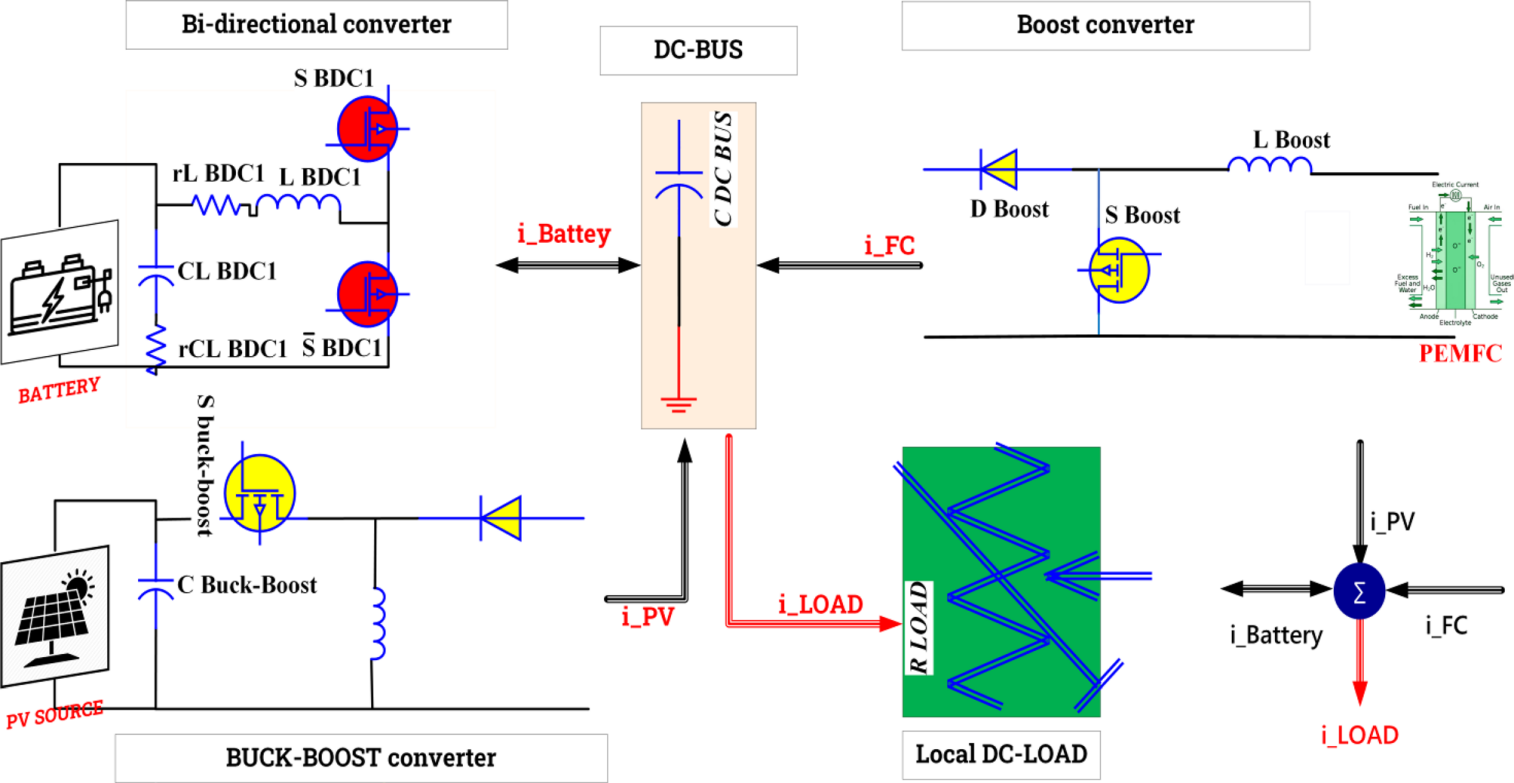
Global system configurations.

The proposed system is represented in Fig. 1 and divided into four parts as follows:

- The photovoltaic system is comprised of photovoltaic panels and a DC/DC converter that is regulated by a fuzzy logic maximum power point tracking (MPPT) algorithm.
- PEMFC is connected to a DC/DC boost converter.
- Batteries are linked together through the utilization of a DC/DC bidirectional converter.
- A DC load.

### Photovoltaic modeling

**Figure 2:**
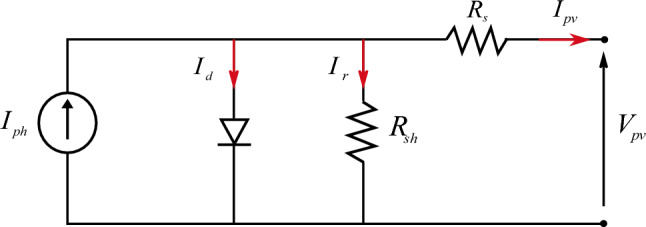
Equivalent model for solar cell.

Photovoltaic (PV) generation is a sustainable energy production method that utilizes photovoltaic cells to convert sunlight into electrical energy through the photovoltaic effect. The P-V and I-V characteristics of the PV array exhibit variability based on external conditions, and a DC/DC converter is employed to connect the PV array to the DC bus for maximum power point tracking (MPPT)^67^. Various MPPT controllers have been proposed by researchers. In reference^68^, the authors proposed a hardware implementation of fuzzy logic control (FLC) through state machine control (SMC) to enhance power output from the photovoltaic array. It is crucial to extract the maximum available power from the photovoltaic array to optimize its performance. In Ref.^69^, a novel fuzzy regression maximum power point tracking (MPPT) method was proposed, and in Ref.^70^, the authors introduced a modified incremental conductance (INC) algorithm for MPPT. Various mathematical models have been developed to illustrate the non-linear behavior of solar panels due to the semiconductor junctions in their design. The PV module is constructed by connecting a series of individual cells in parallel, and the same assembly process is used to create a PV generator or array. The current produced by a single cell can be derived from the electrical circuit illustrated in Fig. 2:

1 $$ I_{pv} = I_{ph} - I_{d} \left[ {\exp (\frac{{q(V_{pv} - I_{pv} R_{s} )}}{nkT}) - 1} \right] - \frac{{V_{pv} + I_{PV} R_{s} }}{{R_{sh} }} $$

From Eq. (1), to make up a PV module, we must connect many PV cells in series (N_s_) and parallel (N_p_); the PV module equation is represented in the equation below:

2 $$ I_{pv} = N_{p} I_{ph} - N_{p} I_{sd} \left( {\exp (\frac{{N_{s} V_{pv} + \left( {N_{s} /N_{p} } \right)R_{s} I_{pv} }}{{nN_{s} KT}}) - 1} \right) - \left( {\frac{{N_{s} V_{pv} + \left( {N_{s} /N_{p} } \right)R_{s} I_{pv} }}{{(N_{s} /N_{P} )R_{sh} }}} \right) $$

The establishment of a connection that links the photovoltaic array and the DC bus is achieved through the utilization of a buck-boost converter, enabling optimal performance; it is imperative for the system to function at its highest energy capacity. The most prevalent type of DC/DC converter is the buck-boost converter, which merges the electrical characteristics of both a buck converter and a boost converter^56^.

**Figure 3:**
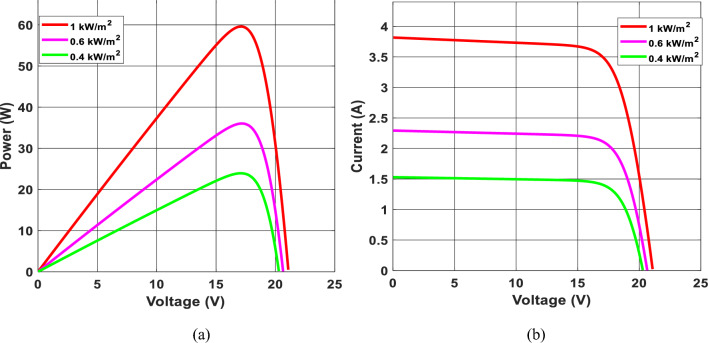
P–V and I–V curves of photovoltaic panel.

Figure 3 depicts a photovoltaic module’s P-V and I-V characteristics under different sun irradiance conditions. As shown in Fig. 3a, as the outcome voltage V_pv_ increases, When the voltage hits a particular threshold, the photovoltaic module’s current changes slightly and soon drops while the output voltage continues to rise^71,72^. As a result, near the highest power limit, the U-I characteristic shapes are non-linear. If the photovoltaic module voltage Vpv remains fixed, the current of the photovoltaic panel Ipv rises proportionately to the increased irradiation. Once the sunlight is constant, PV module power exhibits a non-monotonic tendency with the shift of its V_pv,_ as shown in Fig. 3b. Once the PV voltage is insufficient, the power of the photovoltaic panel’s P_pv_ rises as the output voltage U_pv_ improves^73^. When the photovoltaic module’s Vpv increases, the photovoltaic module’s power Ppv decreases. The P-V characteristic graphs show a significant link between photovoltaic array production power and sunlight. More excellent power adjustment results from a reduced voltage disturbance around the maximum power point.

**Figure 4:**
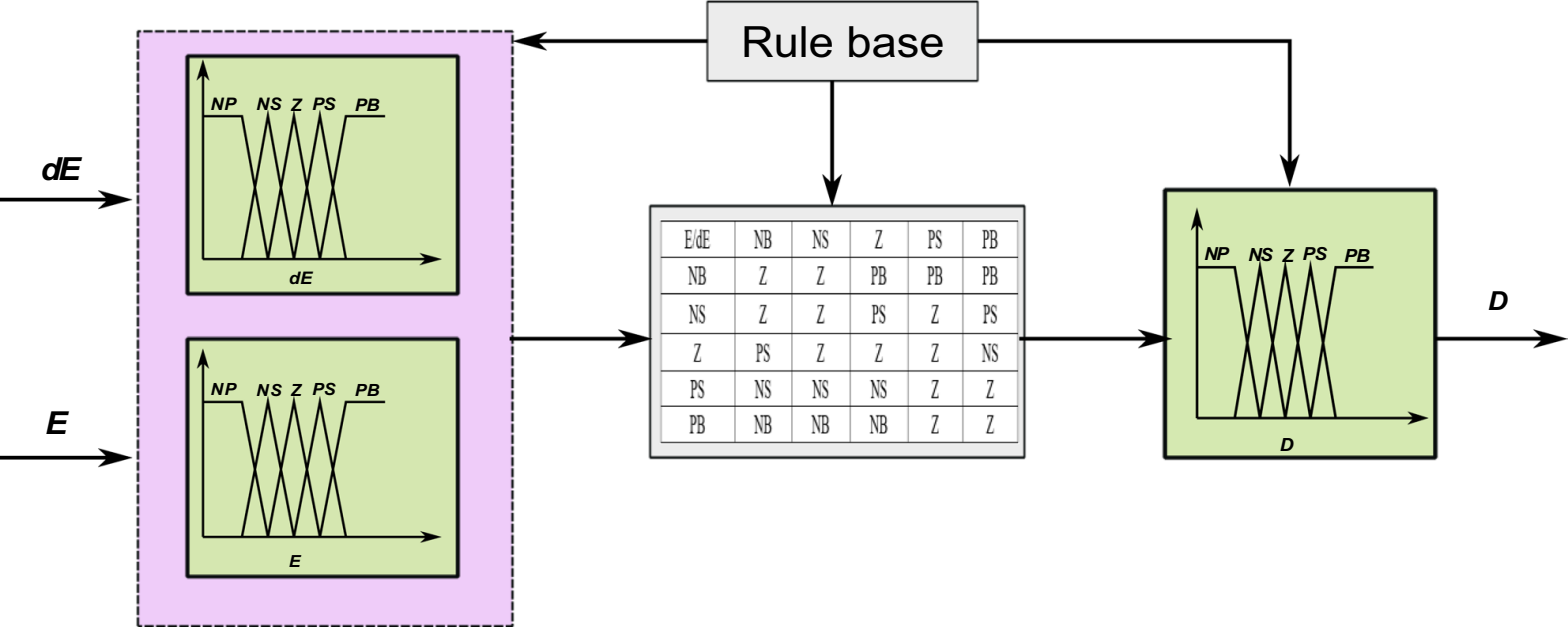
Fuzzy logic membership: E, DE, and D.

The use of PWM simplifies the control of the buck-boost converter. The framework for maximal PowerPoint tracking incorporates the use of fuzzy logic control. Figure 4 illustrates the graphical depiction of the membership functions pertaining to the input variables and the output variable. As shown in Eq. (3), the fuzzy logic controller (FLC) has two inputs and one output: E, DE, and D:

3 $$ \left\{ \begin{gathered} E(k) = \frac{{P_{PV} \left( k \right) - P_{PV} \left( {k - 1} \right)}}{{I_{PV} \left( k \right) - I_{PV} \left( {k - 1} \right)}} \hfill \\ DE\left( k \right) = E(k) - E(k - 1) \hfill \\ \end{gathered} \right. $$

### PEM fuel cell modeling

**Figure 5:**
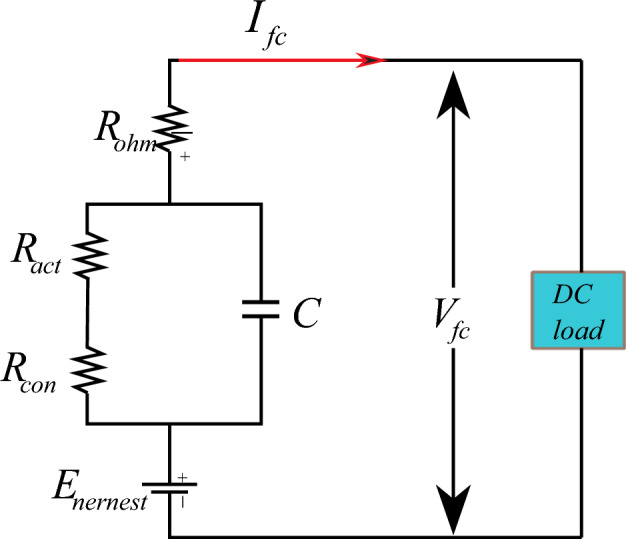
Electrical model of PEM fuel cell.

When compared to other kinds of FCs, the primary benefits of a PEMFC as a power source are adequate electrical power in a steady state, dependable power production, small size, minimal working noise, and eco friendliness^74^. PEMFC is made up of several fuel cells linked in series called stacks; additionally, the PEMFC curve is non-linear and is influenced by temperature, oxygen and hydrogen pressure, and so on. Figure 5 depicts the electrical model of the PEMFC; the voltage of the stack is determined as follows:

4 $$ V_{out} = N.V_{fc} $$

where N represents the total number of PEMFCs associated with the series, and V represents the output voltage.

In general, the output voltage of the fuel cell is represented as follows^75^:

5 $$ V_{fc} = E_{nernst} - v_{act} - v_{conc} - v_{ohmic} $$

The output voltage of the fuel cell mentioned above consists of E_nersent_, V_act_, V_conv_, and V_ohmic_.

6 $$ E_{nernst} = 1.229 - (0.85 \times 10^{ - 3} ) \times T_{FC} - 298.15) + (4.31 \times 10^{ - 5} \times T_{FC} \times \ln (p_{{H_{2} }} ) \times 0.5 \times \ln (p_{{O_{2} }} )) $$

7 $$ v_{act} = - \left[ {\delta_{1} + \delta_{2} T_{fc} + \delta_{3} T_{fc} \ln (C_{{O_{2} }} ) + \delta_{4} T_{fc} \ln (I_{fc} )} \right] $$

8 $$ v_{ohm} = I_{FC} (R_{M} + R_{C} ) $$

9 $$ v_{con} = - \beta \ln \left( {1 - \frac{j}{{j_{\max } }}} \right) $$

E_nersent_ denotes the chemical reactions of output voltage, T_fc_ the temperature, and the P_O2_ and P_H2_ the oxygen and hydrogen levels, respectively. V_act_ represents the activation loss that consists of $\delta_{1,2,3,4}$ empirical coefficients and C_o2_ empirical coefficients. V_ohm_ represents the ohmic voltage that consists of R_M,_ and R_c_ indicates electron flow resistance and proton resistance, respectively. V_con_ donates the concentration overvoltage that consists j, j_max_, and b, representing the maximal current, maximum current density, and concentration loss^76,77^.

**Figure 6:**
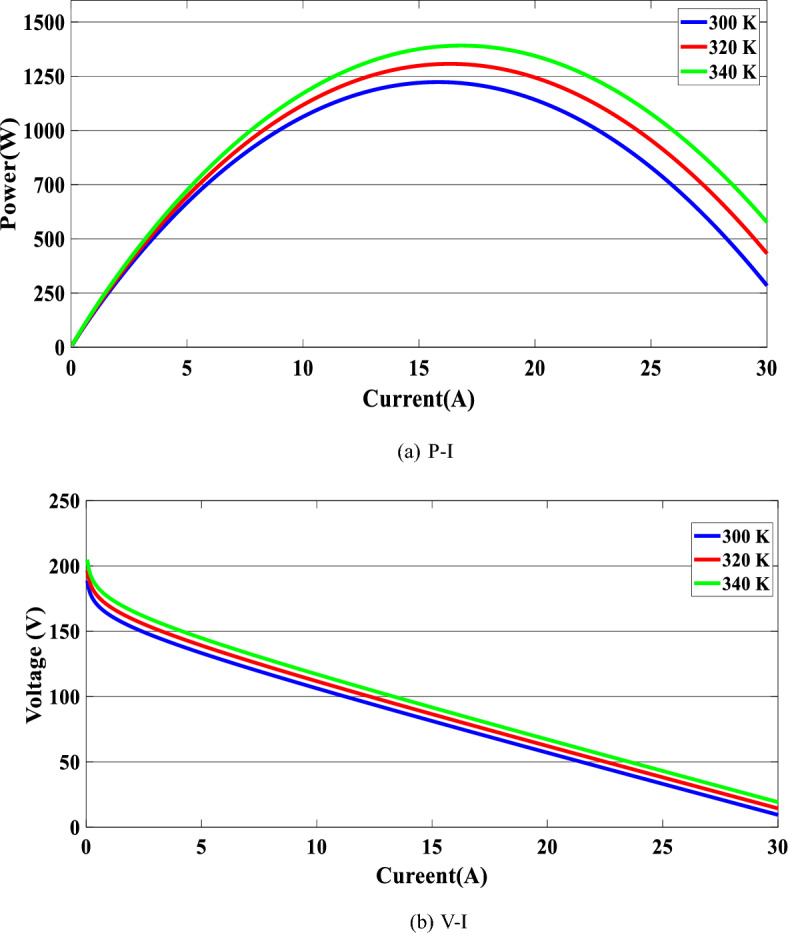
PEMFC characteristic.

Figure 6 below illustrates the properties of a PEMFC stack, specifically its P-V (pressure–voltage) and I-V (current–voltage) characteristics.

P-I and V-I characteristic graphs of the PEMFC stack at various temperatures are shown. As illustrated in Fig. 6a, the increase in voltage and power of the fuel cell when we change the temperature from 300 to 340 K is extremely small. The voltage steadily decreases as the fuel cell current rises, as shown in Fig. 6b.

### Battery storage system

Because of the irregular, random, and unpredictability of renewable energy systems (RES) in loads, implementing a BES is critical to supporting energy output and dealing with RES breakdowns^78,79^. This study applies lithium ion battery technology to a collection of DC-load units. Lead-acid (LA) batteries are an established technology that is suited for static uses and has a cheap initial expenditure cost. Lithium batteries, on the other hand, have better energy and power densities, greater performance, an extended life span, and other technical advantages over competing kinds^80,81^. As a result of their outstanding performance, these types of batteries are also well-suited for fixed solar systems and the provision of uninterrupted electrical power^82,83^. To choose the best battery type, a number of technological factors are taken into account, including maturity, weight, size, discharge rate, temperature sensitivity, upkeep, and the effectiveness of costs^84,85^. In this paper, we use the lithium-ion battery for the advantages mentioned above and summarized in Table 1 below, and the supplementary appendix shows the statistics for the Li-ion battery, the DC/DC boost converter, and the buck converter’s specifications.

**Table 1:** Comparison between lithium-ion and lead acid^70^.

Lithium-ion	Lead acid
Efficiency (%)	
85–95%	60–80%
Advantages	
Low upkeep, high effectiveness, lightweight, and low self-discharge	Low capital cost, grown technology
Drawbacks	
More susceptible to high temps, and is still regarded as a developing technology	The maintenance includes watering, and watching the 50% flow limit—inadequate effectiveness

### Power converter

**Figure 7:**
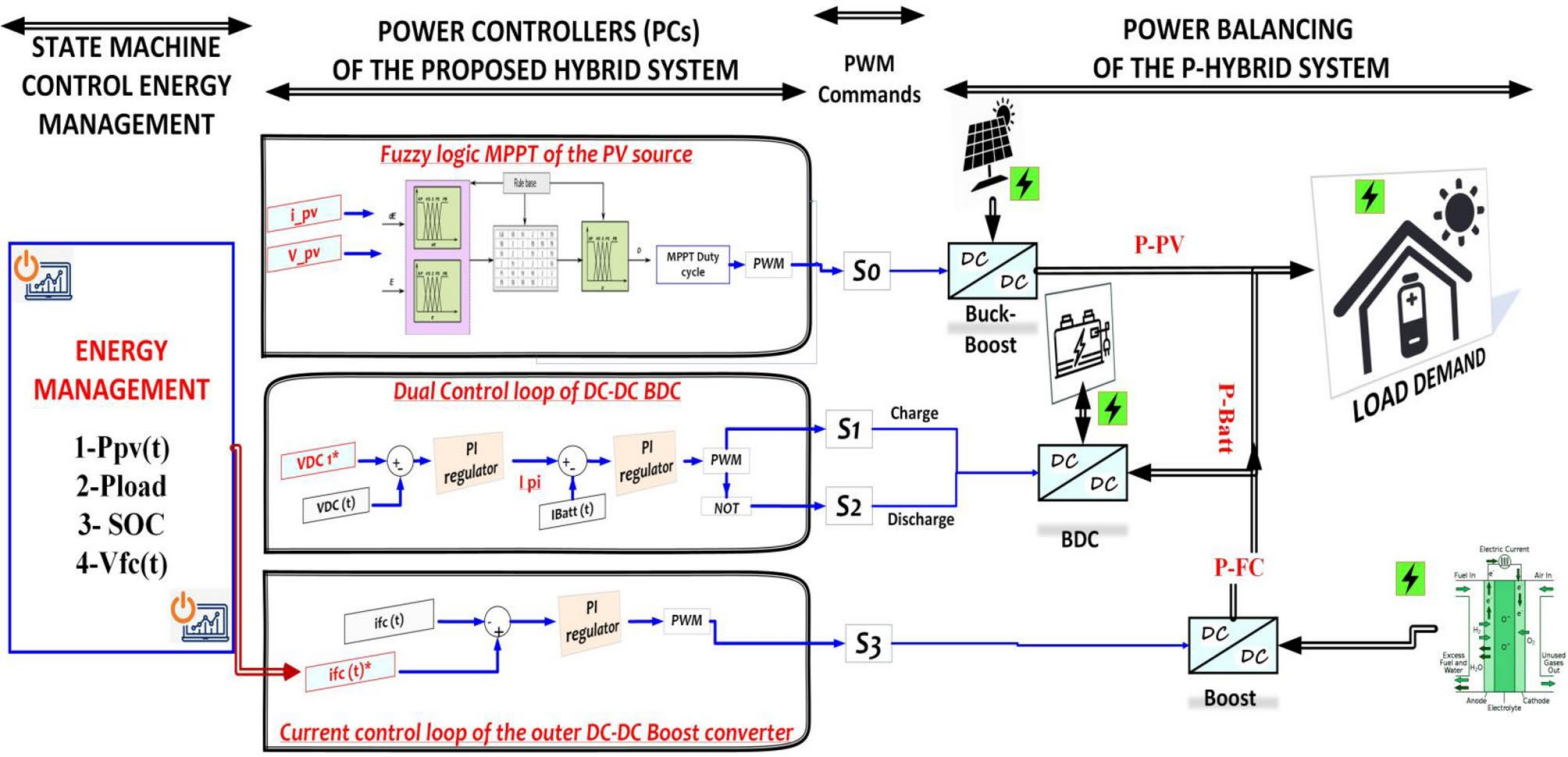
State machine energy management, power controller, and power balancing of the proposed system.

The primary objective of this research is to examine non-isolated DC-DC converters that establish a connection between PV, PEMFC, and battery systems and the load through a DC-coupled bus, as depicted in Fig. 7. The buck-boost converter is a unidirectional apparatus that ensures the flow of I_pv_ for the purpose of either charging the battery or distributing the load through the local DC bus^86^. The boost converter provides the provision of the proton exchange membrane fuel cell (PEMFC) current to the load through the direct current (DC) bus. Additionally, it has the capability to charge the battery if necessary. The bi-directional converter (BDC) ensures the bidirectional transfer of electrical current between the battery and the load through the direct current (DC) bus^82,87^. Figure 7 illustrates the power control scheme of the hybrid system under consideration. The DC-link voltage reference, the reference current for the PEM Fuel Cell (PEMFC), and the reference current for the battery are denoted as V^*^DC, I^*^fc, and I^*^batt, respectively. The photovoltaic (PV) power is monitored and regulated using a buck-boost converter while being controlled by a fuzzy logic maximum power point tracking (MPPT) algorithm under varying environmental conditions^88,89^. The BDC employs a dual-loop configuration to effectively identify the DC-link voltage and subsequently regulate it in order to compensate for any power imbalances through the utilization of battery storage charge and discharge mechanisms^90,91^. The calculation of load power is determined by the equation provided below:

10 $$ p_{load} = V_{dc - bus} .I_{dc - bus} = V_{dc - bus} C_{dc - bus} .(V_{dc - bus} /dt) $$

It has been proposed that the DC link is a capacitor. Once all of the basic models were defined, we coupled the power sources according to the DC bus design. Direct current (DC) is provided by every supply source gathered on the bus. The calculation for the DC link current is as follows:

11 $$ C_{dc - bus} \frac{{dV_{dc - bus} }}{dt} = I_{pv} + I_{fc} \pm I_{batt} $$

The efficiency of the global system (converters and the load) is represented by *η* and could be expressed in the equation below:

12 $$ P_{load} = P_{pv} \eta_{buck - boost} + P_{fc} \eta_{boost} + P_{pv} \eta_{BDC} $$

13 $$ \eta_{load} = (\eta_{pc} + \eta_{fc} + \eta_{BDC} )/3 $$

## Suggested state machine energy management strategy

This section centers on the formulation of a power control strategy for an integrated system. An innovative home energy management system (HEMS) has been devised to monitor and regulate the efficiency of the proposed system. The operational strategy designed for this system must account for fluctuations in solar irradiation. The calculation of the net power for the proposed hybrid system can be formulated as follows:

14 $$ P_{net} = P_{load} - P_{pv} $$

One of the control strategies that is based on rules for microgrids is the state machine control strategy for this proposed hybrid system. As shown in Table 2, the state machine control method used comprises 15 states under three SOC intervals. The three different SoC intervals are classified as high means that (SOC > SOC_max_), Normal means that (SOC_max_ > SOC < SOC_min_); and Low means that (SOC < SOC_min_). The highest limit SOC_max_ and lower limit SOC_min_ of the SOC. It is clear that the batteries' SOC interval, besides the Net power of the system P_net_ used to calculate the PEMFC reference power and concurrently taken into consideration, are the following limits: Ppv is the output power of the PV generation device, and Pload is the load requirement; PEMFC system’s minimal, optimal medium and maximum output powers are designated as Pfc_min_, Pfc_opt_, P_mid_, and Pfc_max_ respectively, the SOC_max_ and SOC_min_ represent the minimal and the maximal limits respectively.

**Table 2:** Proposed state machine control.

State	SOC	p_net_ (t)	P*_fc_ (t)
1	Low	p_net_ (t) < Pfc_min_	P*_fc_ = Pfc_opt_
2		p_net_ (t) $\in$ [Pfc_min_,Pfc_opt_[	P*_fc_ = Pfc_opt_ + Pfc_min_
3		p_net_ (t) $\in$ [Pfc_cop_,Pf_mid_[	P*_fc_ = P_mid_
4		p_net_ (t) $\in$ [Pfc_mid_,Pfc_max_[	P*_fc_ = Pfc_mid_ + Pfc_opt_
5		p_net_ (t) ≥ Pfc_max_	P*_fc_ = Pfc_max_
6	Medium	p_net_ (t) < Pfc_min_	P*_fc_ = Pfc_min_
7		p_net_ (t) $\in$ [Pfc_min_,Pfc_opt_[	P*_fc_ = Pfc_min_ + Pfc_opt_
8		p_net_ (t) $\in$ [Pfc_cop_,Pf_mid_[	P*_fc_ = Pfc_opt_
9		p_net_ (t) $\in$ [Pfc_mid_,Pfc_max_[	P*_fc_ = P_net_
10		p_net_ (t) ≥ Pfc_max_	P*_fc_ = Pfc_mid_ + Pfc_min_
11	Hight	p_net_ (t) < Pfc_min_	P*_fc_ = Pfc_min_
12		p_net_ (t) $\in$ [Pfc_min_,Pfc_opt_[	P*_fc_ = Pfc_mid_ − Pfc_opt_
13		p_net_ (t) $\in$ [Pfc_cop_,Pf_mid_[	P*_fc_ = Pfc_opt_
14		p_net_ (t) $\in$ [Pfc_mid_,Pfc_max_[	P*_fc_ = Pfc_mid_ − Pfc_min_
15		p_net_ (t) ≥ Pfc_max_	P*_fc_ = Pfc_mid_

## Results and discussions

**Figure 8:**
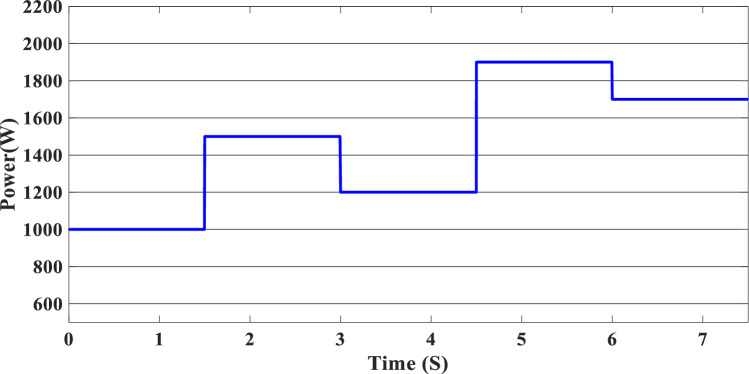
Load demand profile.

**Figure 9:**
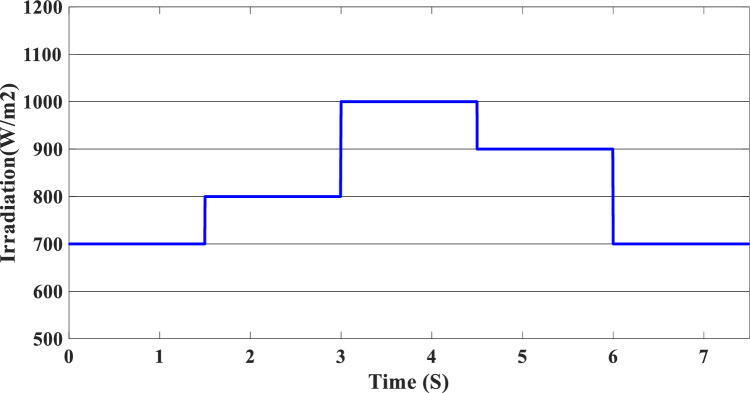
Irradiation profile.

The study of the suggested state machine control for the proposed hybrid system is shown in the section that follows using the MATLAB/Simulink (version: 2016, link: https://in.mathworks.com/products/simulink.html) toolbox. The supplementary appendix displays the global hybrid system’s parameters. The findings of the scenario study emphasize the impact of using variable irradiation for the PV source and the system’s capacity to function effectively under high loads, which is significantly influenced by the different SOC levels. The findings are presented sequentially: PV-PEMFC-battery powers and Load power waveforms, DC bus voltages, and SOCs of the battery, and finally efficiency of the global system in different cases. In this case, the temperature stays at 25 ℃ while the irradiation ranges from 700 to 1000 watts per square meter; we divide this scenario into three cases relying on the SOC first case SOC > SOC_min_ (low), second case SCO_min_ < SOC < SOC_max_ (medium) and third case SOC > SOC_max_ (hight) and the load-interval of the load = [1000 W–1900 W]. The load demand profile is shown in Fig. (8), the maximum power demand is equal to1900 W, and the minimum value is equal to 1000 W and is divided into time intervals as following:1000 W ∈ [0 s–1.5 s] 1500 W ∈ [1.5 s–3 s], 1200 W ∈ [3 s–4.5 s], 1900 W ∈ [4.5 s–6 s] and 1700 W ∈ [6 s–7.5 s] illustrated in Fig. 8. As represented in Fig. 9, the irradiation profile is divided in the interval time as follows: 700 W ∈ [0 s–1.5 s] 800 W ∈ [1.5 s–3 s], 1000 W ∈ [3 s–4.5 s], 900 W ∈ [4.5 s–6 s] and 700 W ∈ [6 s–7.5 s].

### Case 1: SOC < SOC_min_

**Figure 10:**
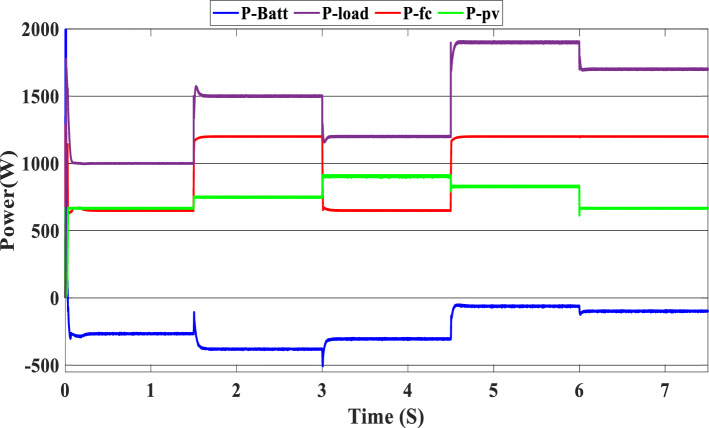
Power generated by the hybrid system (SOC < SOC_min_).

Figure 10 illustrates the variations in power forms for the PV, PEMFC, and batteries, as well as the fluctuations in power demand for the hybrid generating system. The solar system’s maximum power output is 903 W, while the PEMFC’s maximum output is 1350 kW.

At the time interval [0 s–1.5 s], the PV irradiation is equal to 800 W/m^2^, and the maximum power is equal to 670 W, which is less than the load demand, at the same time to reaches 1000 W which is the load demand, the PEMFC system generated 646 W in order to fulfill the required load demand power and charge the batteries at the same time because the SOC of the batteries is lower than the SOCmin which is equal to 20%. Then, the time interval is equal to [1.5 s–3 s], and the load demand is equal to 1500 W. To reach this value, the irradiation of the PV system is equal to 800 W/m^2^ and generates power equal to 743 W; the current value is inadequate to fulfill the required load; in this case, the PEMFCs generate power equal to 1.197 kW in order to fulfill the requirements of the load and facilitating the battery charging procedure.

**Figure 11:**
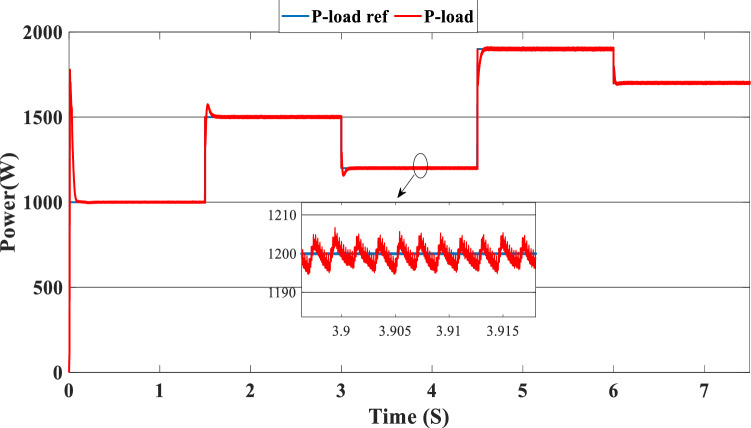
Load power generated from the hybrid system (SOC < SOC_min_).

The PV system produces 903 W at 1000 W/m^2^ from 3 to 4.5 s, requiring power higher than the PV power and equal to 1200 W. In this case, the PEMFC generated a power = 646 W. This power can fulfill the necessary power of load and the surplus power to be utilized for charging the batteries. At 4.5 s, the demand power is high and equal to 1900 W, which is higher than the PV system power, equals 832 W, which means we need more power, so the requirement for power is derived from the PEMFC system, which is equal to 832 W, and the excess power goes to the batteries directly to charge them. Finally, in the time interval [6 s–7.5 s], the load demand is 1700 W, which is greater than the maximum power of the PV system, which means that the PEMFC generates power equal to 1.197 kW to meet needed power needed in the load and charges the batteries. Figure 11 represents the reference load and the load power generated by the hybrid system. It can be seen that the generated power follows the reference load; this implies the effectiveness of the suggested SMC.

**Figure 12:**
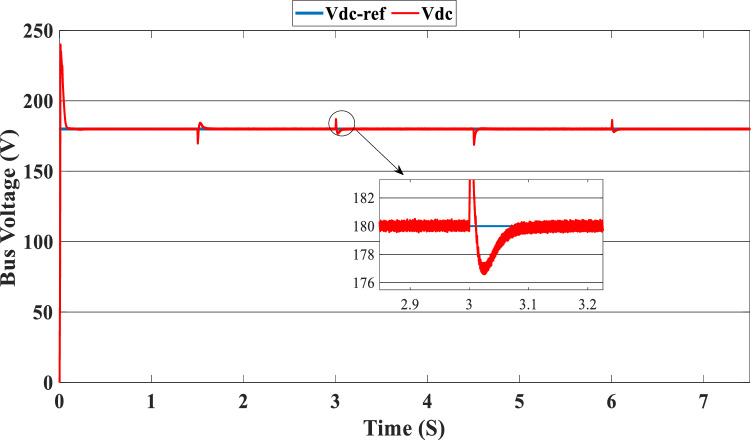
DC bus voltage (SOC < SOC_min_).

**Figure 13:**
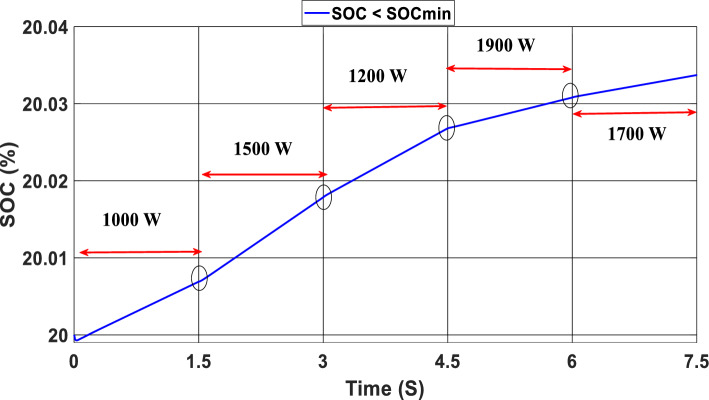
State of the batteries (SOC < SOC_min_).

**Figure 14:**
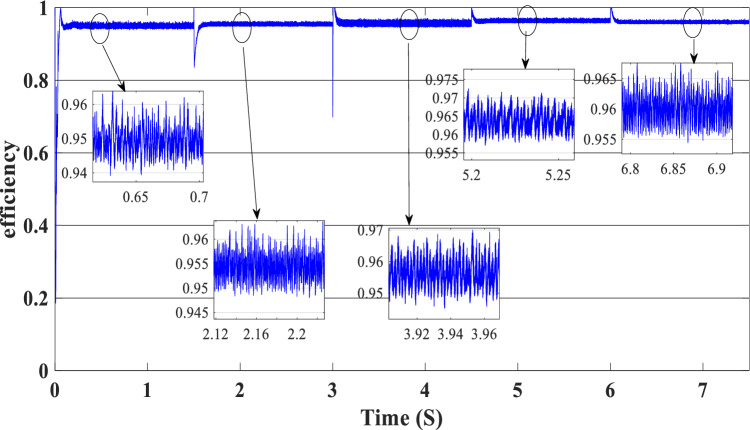
Efficiency of the global hybrid system (SOC < SOC_min_).

Figure 12 illustrates the DC link voltage, the reference value equal to 180 W. We noticed that the bus voltage tracks the reference efficiently; the picks in the 1.5 s, 3 s, 4.5 s, and 6 s are caused by the change in the load demand. We see there is a deviation from the reference and return rapidly to track the reference value. Figure 13 represents the SOC of the batteries; we notice that the batteries, in this case, are charging no matter what the value of the load demand; the power generated from the PEMFC system supplies the power to the load and charges the battery because the SOC of the batteries is less than the SOC_min_. Figure 14 depicts the calculated efficiency of the globe hybrid (buck-boost converter, boost converter, and bi-directional DC/DC converter) during the change in irradiation. This is the case when the SOC is lower than the SOC_min_. Moreover, the efficiency of the hybrid system is summarized in Table 3 below.

**Table 3:** Efficiency of the global system (SOC < SOC_min_).

Time	Load demand (W)	Efficiency (%)
[0 s–1.5 s]	1000	95
[1.5 s–3 s]	1500	96
[3 s–4.5 s]	1200	95.5
[4.5 s–6 s]	1900	96
[6 s–7.5 s]	1700	96

### Case 2: SOC_min_ < SOC < SOC_max_

**Figure 15:**
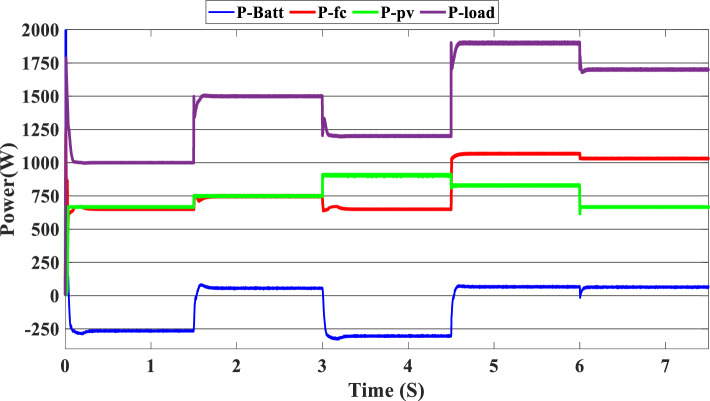
Power generated by the hybrid system (SOC_min_ < SOC < SOC_max_).

In this case, we suppose the SOC = 65%, which means it’s between SOC_max_ and SOC_min_. As shown in Fig. 9, the PV irradiance profile starts from 700 to 1000 W/m^2^ with a sudden change in 1.5 s, 3 s, 4.5 s, and 6 s, respectively. The variation in load power demand mentioned in Fig. 15 shows the power produced by the PV system, PEMFC system, and storage. Additionally, when the demand is 1000 W, and the PV system is producing 670 W, this indicates that we need more power to carry out the load because this number is less than the load requirement. In this instance, the PEMFC system produces a power of 646 W, which is more than enough to carry out the load and fully charge the batteries. In the following time interval [1.5 s–3 s], the PV’s irradiation changes from 700 to 800 W/m^2^, the PV system produces more power equal to 754 W, and at the same time, the load demand increases to 1500 W, the current level of supply is inadequate to satisfy the existing demand. In this case, the PEMFC and the batteries generate power 740 W from the PEMFC and 62 W from the batteries). At 3 s, the power of the load decreased to 1200 W, and the PV generates power equal to 912 W/m^2^ while the irradiation is equal to 1000 W; we need more energy to meet the load power, in this case, the PEMFC power is equal to 646 W and it’s enough and the exceed power go to charge the batteries. The load demand increased to 1900 W during the following time frame [4.5 s–6 s], which indicates that neither the PV nor the PEMFC could meet the load’s requirements. In the present scenario, the batteries generated sufficient power to fulfill the load's energy requirements.

The precise duration range is [6 s–7.5 s]. The PV system cannot satisfy the requirements when the load is reduced to 1700 W, and the sunlight is equivalent to 700 W/m^2^, so the PEMFC and batteries generate the necessary power, as shown in Fig. 15.

**Figure 16:**
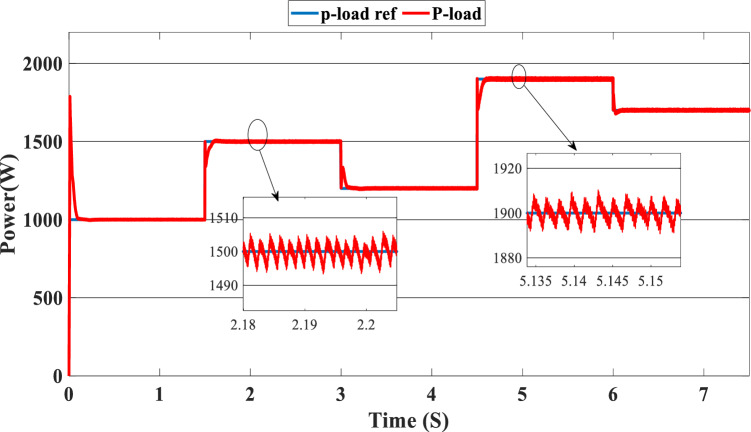
Load demand power (SOC_min_ < SOC < SOC_max_).

**Figure 17:**
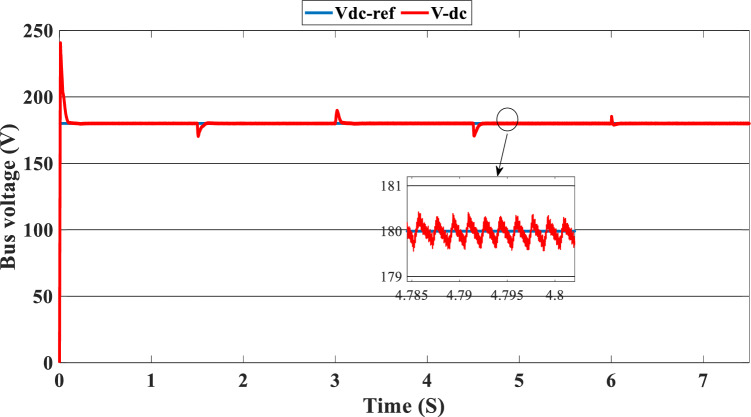
DC-bus voltage (SOC_min_ < SOC < SOC_max_).

**Figure 18:**
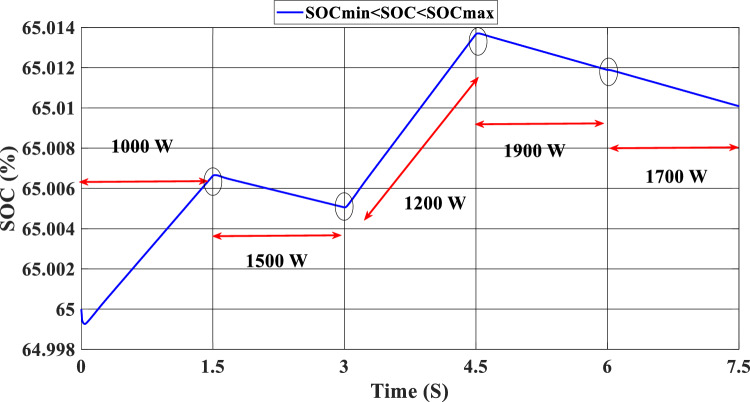
State of the batteries (SOC_min_ < SOC < SOC_max_).

**Figure 19:**
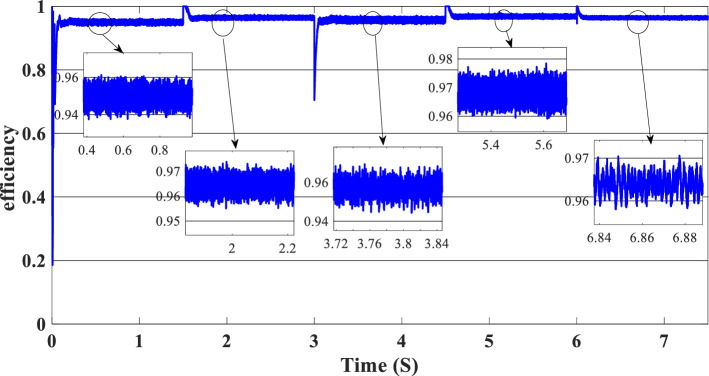
Efficiency of the hybrid system (SOC_min_ < SOC < SOC_max_).

Figure 16 illustrates the required load. As can be seen, the total power generated by the PV, PEMFC, and batteries tracks the reference load power, indicating that the suggested SMC operates effectively. Figure 17 shows the DC-link voltage; as we can see, it follows the 180 V reference voltage. However, we also observed that the bus voltage deviates from the reference when the load demand changes and we quickly return to tracking the reference voltage with a short response time. Figure 18 represents the SOC of the batteries in intervals of time [0 s–1.5 s]. The batteries charge because there is an exceeded power from the (PV and PEMFC), next from 1.5 to 3 s, the batteries discharge because the PV+PEMFC can't satisfy the load demand, so the battery gives a power, [3 s–4.5 s] the batteries charge finally in the interval time [4.5 s–7.5 s]. The batteries discharge no matter the load power value because we need the power to fulfill the needs of the load. Figure 19 represents the efficiency of the suggested system; in this case, the SOC belongs to the interval SOC_min_ and SOC_max_; from the figure, we can notice that the lowest efficiency is 95%, and the maximum efficiency is 97%, the Table 4 below summarized the results founds.

**Table 4:** Efficiency of the global system (SOC_max_ < SOC < SOC_max_).

Time	Load demand (W)	Efficiency (%)
[0 s–1.5 s]	1000	95
[1.5 s–3 s]	1500	95.5
[3 s–4.5 s]	1200	95.5
[4.5 s–6 s]	1900	97
[6 s–7.5 s]	1700	96.5

### Case 3: SOC > SOC_max_

**Figure 20:**
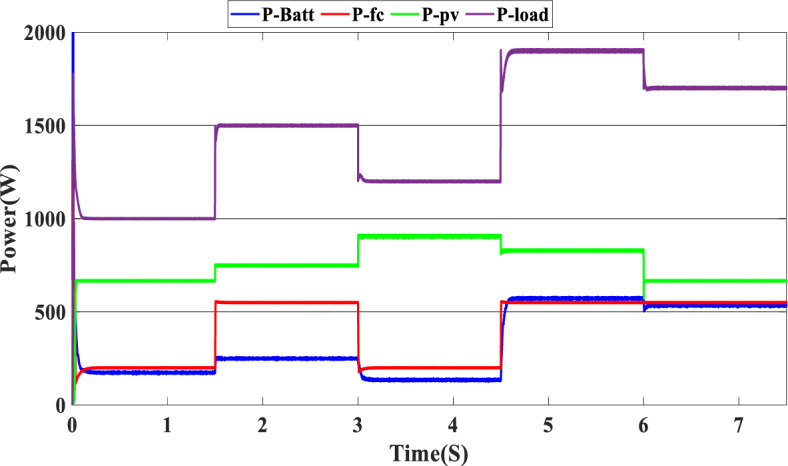
Power generated by the hybrid system ( SOC > SOC_max_).

**Figure 21:**
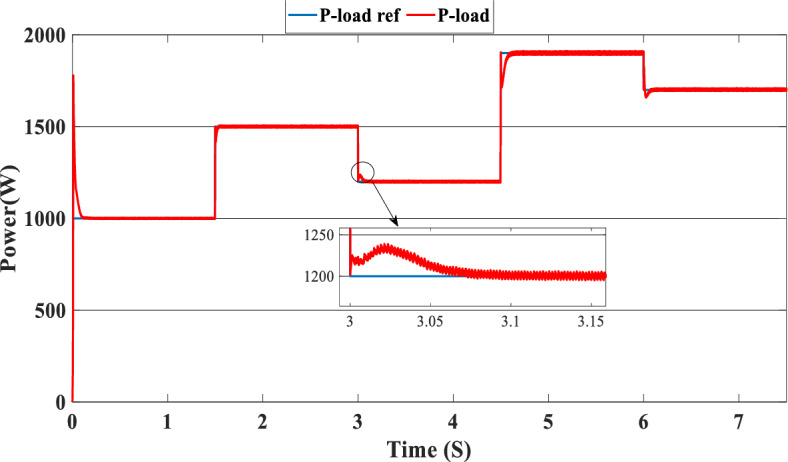
Load demand (SOC > SOC_max_).

**Figure 22:**
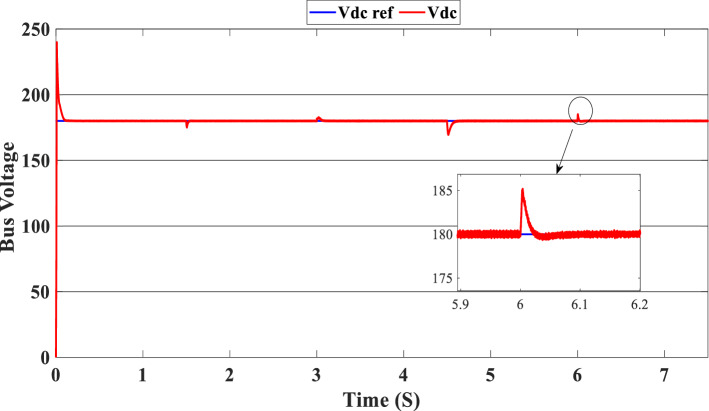
DC link voltage (SOC > SOC_max_).

**Figure 23:**
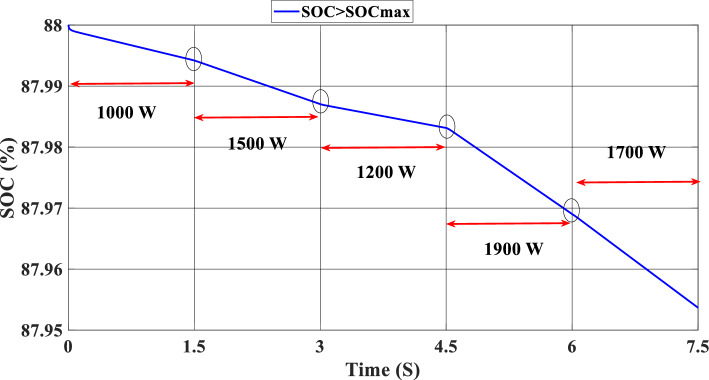
State of the batteries (SOC > SOC_max_).

**Figure 24:**
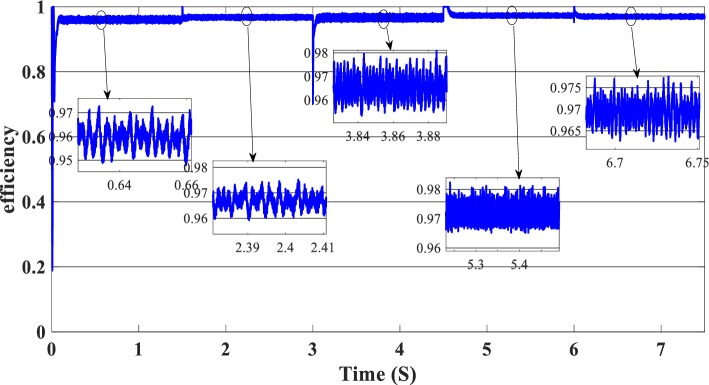
Efficiency of the hybrid system (SOC > SOC_max_).

In this case, the state of the charger is higher than SOC_max,_ which is equal to 88%, to test the objective of the global proposed system like the previous cases at the same irradiation profile and the load demand. Figure 20 illustrates the power output derived from the renewable hybrid source alongside the consumption of the load. The power in the load is always higher than the primary source (PV); in the first interval [0 s–1.5 s], the PV system generates power not enough to fulfill the load demand; in this case, the batteries generate power to meet the needs, and the PEMFC also generates a minimum power to support the batteries. From 1.5 s, the irradiation change to 800W/m^2^ the power generated from (PV) is less than the required load this case, like the previous one, the batteries still discharge, and the PEMFC supports the needs of the power needs when the (PV+Batteries) cant meets the need of the Load, now comes the interval time [3 s–4.5 s] in this time the load demand 1200 W which means the PV+batteris+PEMFC must generate power to fulfill the load and that is what we can see from the figure, that the batteries +PV generate a power to fulfill the load and the PEMFC work in a lower value to support the needs. When the irradiation is equal to 900 W/m^2^, in the time interval [4.5 s–6 s], the PV produces power, and the batteries also discharge, and the need for the power to fulfill the load is compensated by the PEMFC. Finally, when the interval time [6 s–7.5 s], the power from the primary source (PV) produces low power in the previous state the batteries are still discharging, and the PEMFC gives the exact value of the power to complete The photovoltaic system (PV), in conjunction with batteries, fulfill the load demand, which is equivalent to 1700 W. Figure 21 represents the load demand. We can see that the load follows the reference load with a small time response when we change the load value. Figure 22 depicts the DC link voltage. DC bus voltage follows the standard 180 V, with deviations occurring every 1.5 s, 3 s, 4.5 s, and 6 s. Figure 23 represents the SOC of the batteries with the initial value equal to 88% > SOC_max_. In this case, the batteries must discharge whatever the value of the load and the primary source is. Figure 24 summarizes the global system’s effectiveness for different load demand values in Table 5 below.

**Table 5:** Efficiency of the global system (SOC > SOC_max_).

Time	Load demand (W)	Efficiency (%)
[0 s–1.5 s]	1000	96
[1.5 s–3 s]	1500	96.5
[3 s–4.5 s]	1200	96.5
[4.5 s–6 s]	1900	97.5
[6 s–7.5 s]	1700	97

## Conclusion and future research directions

This study introduced a new state machine control (SMC) for a multi-source renewable system (MSRS) that includes photovoltaic (PV), proton exchange membrane fuel cell (PEMFC), and batteries. The proposed control method was assessed using MATLAB/Simulink (version: 2016, link: https://in.mathworks.com/products/simulink.html) simulations. The SMC strategy was devised to efficiently oversee the energy resources in the MSRS, taking into account fluctuating solar irradiation levels and battery state of charge (SOC). The simulation results showcased the resilience and efficacy of the suggested SMC in regulating the MSRS across different operational circumstances. The MSRS was determined to effectively fulfill the load requirement while simultaneously ensuring stability and achieving satisfactory performance. The system closely monitored and controlled key parameters such as DC bus voltage, battery state of charge (SOC), and overall system efficiency, ensuring they remained within specified limits. In summary, this research contributes to the advancement of efficient energy management techniques for renewable energy systems, emphasizing the importance of optimal power control strategies in enhancing the overall performance and sustainability of MSRS. However, further experimental validation is required to corroborate the simulation findings and ensure real-world applicability. Additionally, future research could explore integrating the MSRS with grid systems to enable the bi-directional flow of excess electricity, further enhancing the system's flexibility and reliability in meeting dynamic energy demands. This study paves the way for the development and deployment of innovative energy management strategies for a more sustainable and environmentally friendly future.

Future research endeavors in this domain may focus on addressing the reliability and robustness of the state machine control (SMC) strategy under varying conditions, notably through real-world testing to verify the performance of the multi-source renewable system (MSRS). Further exploration could also involve the integration of the MSRS with the grid to enable energy exchange and optimize renewable resource utilization, while potential synergies and trade-offs between different energy sources, such as wind, hydropower, and solar, could be studied for the development of more efficient and reliable hybrid energy systems. There’s also a strong potential in developing more advanced energy management algorithms, such as machine learning-based approaches, to enhance the system’s adaptability and efficiency. Furthermore, an environmental impact assessment could be performed to compare the system’s sustainability and carbon footprint to traditional energy systems, while a full economic study can determine the system’s cost-effectiveness and return on investment. Furthermore, the creation of supportive legislative frameworks and regulatory standards may pave the road for widespread implementation. Finally, by studying these pathways, future research can considerably develop multi-source renewable systems while also promoting sustainable and resilient energy solutions. PEMFC is a reliable and durable backup power supply in the given scenario. It also charges the batteries and acts as the third backup option when the energy sources fail to satisfy the load requirements. To optimize future operations, it is recommended to construct a direct link between this system and the network to streamline the process of injecting surplus power. We will also add electrolysis to complete the global system.

### Supplementary Information

Supplementary Information.

## Data Availability

The datasets used and/or analysed during the current study available from the corresponding author on reasonable request.
