# Supplementary material for: Advanced efficient energy management strategy based on state machine control for multi-sources PV-PEMFC-batteries system
Source: Sci Rep. 2024 Apr 5;14:7996. doi: 10.1038/s41598-024-58785-2 (PMC10997768; doi:10.1038/s41598-024-58785-2)
Supplement: Supplementary file 1 — Supplementary Information. [file 41598_2024_58785_MOESM1_ESM.docx]

**APPENDIX**

| **PV Module** | **Value** |
| --- | --- |
| Max poawer | 60 w |
| Max voltage | 17.1 V |
| Max current | 3.5 A |
| Series modules | 5 |
| Parallel modules | 3 |
| **PEMFC** | **Value** |
| A | 70cm^2^ |
| ε_1,_ ε_2_ ε_3_ ε_4_ | -0.944,0.0354,7.5 x 10^-8^,1.96x 10^-4^ |
| Rc | 15x10^-4^ |
| Jmax | 1.7A/cm^2^ |
| **BATTERY** | **Value** |
| Number of series battery 12v | 8 |
| Nominal voltage | 96 V |
| Rated capacity | 14 Ah |
| **Converters parameters** | **Value** |
| C_dc_ | 1100 x10^-6^ F |
| **Buck boost** |  |
| C_in_ | 1 x10^-4^ F |
| L | 40 x 10^-3^ H |
| **Boost** |  |
| L | 0.05 H |
| **Bi-directional** |  |
| C_in_ | 1 x 10^-4^ F |
| L | 1 x 10^-2^ H |
